# Daratumumab and venetoclax in combination with chemotherapy provide sustained molecular remission in relapsed/refractory CD19, CD20, and CD22 negative acute B lymphoblastic leukemia with *KMT2A-AFF1* transcript

**DOI:** 10.1186/s40364-021-00343-3

**Published:** 2021-12-20

**Authors:** Sophie Voruz, Sabine Blum, Laurence de Leval, Jacqueline Schoumans, Françoise Solly, Olivier Spertini

**Affiliations:** 1 Service and Central Laboratory of Hematology, Lausanne University Hospital (CHUV), Lausanne, Switzerland; 2 Service and Central Laboratory of Hematology, Centre Hospitalier Universitaire Vaudois and University of Lausanne, CH-1011 Lausanne, Switzerland; 3 Lausanne University (UNIL), Lausanne, Switzerland; 4 Institute of Pathology, Department of Laboratory Medicine and Pathology, Lausanne University Hospital and Lausanne University, Lausanne, Switzerland; 5 Oncogenomics laboratory, Lausanne University Hospital (CHUV), Lausanne, Switzerland

**Keywords:** Relapsed/refractory B-cell precursor acute lymphoblastic leukemia, Adult B-cell lymphoma/leukemia, Targeted treatment, Chemotherapy regimen, Daratumumab, CD38, venetoclax, Bcl-2, Refractory disease, Immunotherapies

## Abstract

Relapsed/refractory (R/R) B-cell acute lymphoblastic leukemia (B-ALL) has a very poor prognosis with a median overall survival of four to nine months. Achieving a complete molecular response is most often required to obtain a sustained leukemia-free survival after allogeneic hematopoietic stem cell transplantation. Immunotherapies targeting CD19, CD20, or CD22 are very efficient in achieving this goal. However, in the absence of the expression of these immunotherapeutic targets by lymphoblasts, treatment options are extremely scarce. We report the successful treatment of a 26-year-old man who suffered R/R, CD19, CD20, and CD22 negative B-ALL targeting Bcl-2 and CD38 by combining venetoclax and daratumumab with chemotherapy.

**Supplementary Information:**

The online version contains supplementary material available at 10.1186/s40364-021-00343-3.

To the editor.

Patients with R/R B-ALL have unmet clinical needs [1, 2]. Achieving a complete molecular response is most often required to obtain a sustained leukemia-free survival after allogeneic hematopoietic stem cell transplantation (ASCT). Immunotherapies targeting CD19 or CD22 are very efficient in achieving this goal. However, B-ALL may escape targeted therapies by developing CD19 or CD22 negative blast cells and/or by switching to a mixed myeloid and lymphoid phenotype.

A 26-year-old man presented with asthenia and fever. The white blood cell count was 121 G/l, hemoglobin 65 g/l, and platelets 44 G/l. 95% of bone marrow cellularity was infiltrated by a pro-B ALL that was positive for CD34, HLA-DR, CD19, CD38, TdT, BCL-2, partially CD79a, and negative for CD10, CD123, cytoplasmic IgM and cytoplasmic CD3. Conventional karyotype was normal (46,XY). Polymerase chain reaction (PCR) and fluorescence in situ hybridization detected a *KMT2A-AFF1* fusion gene transcript. The ALL was treated according to the GRAALL-2005 induction protocol [3]. A morphologic complete remission (CR) was achieved on Day39. However, minimal residual disease (MRD) was positive with both real-time quantitative PCR of *KMT2A-AFF1* fusion transcript (11% of *ABL* reference gene) and allele specific real-time quantitative PCR of immunoglobulin heavy chain (IGH) gene rearrangement (10^-2^ relative to the diagnostic sample). Accordingly, the ALL was considered at very high risk and an ASCT was planned. Despite two high dose cytarabine and methotrexate consolidation chemotherapies [3], the response remained insufficient with positive MRD on Day82 (*KMT2A-AFF1*: 2.67%). Blinatumomab (15 mcg/m2/d) was started as a bridge to ASCT from an HLA-identical sibling donor. Despite 3 cycles of blinatumomab, pre-transplantation MRD, an independent major risk of relapse [4], remained unfavorable (*KMT2A-AFF1* 0.012% and IGH 10^-3^). ASCT was performed on Day203 after a myeloablative conditioning (total body irradiation, cyclophosphamide, and etoposide) without T-cell depletion. Post-transplant MRD was undetectable for a year. Unfortunately, MRD became positive at Day582 (IGH 7 × 10^-4^) (Fig. 1). Despite one cycle of blinatumomab followed by donor lymphocyte infusion (DLI), MRD progressively increased, without additional genetic mutations. The B-ALL escaped the blinatumomab monotherapy following the loss of CD19 expression by lymphoblasts (Fig. 2.1). Despite FLAG-Ida salvage therapy, and two additional DLIs, ALL relapsed 5 months later (IgH MRD: 7 × 10^-1^ and *KMT2A-AFF1* 40.6% on Day826; DNA microarray identified the deletion of 11q23.3q23.3 involving KMT2A gene with a frequency of 80% and KMT2A rearrangement was detected by FISH at the frequency of 47%). At that time, the blast cell immunophenotype was as follow: CD19-, CD200+, CD10-, CD20-, CD38+, CD22-, CD81+, CD34+, CD117-.

**Fig. 1:**
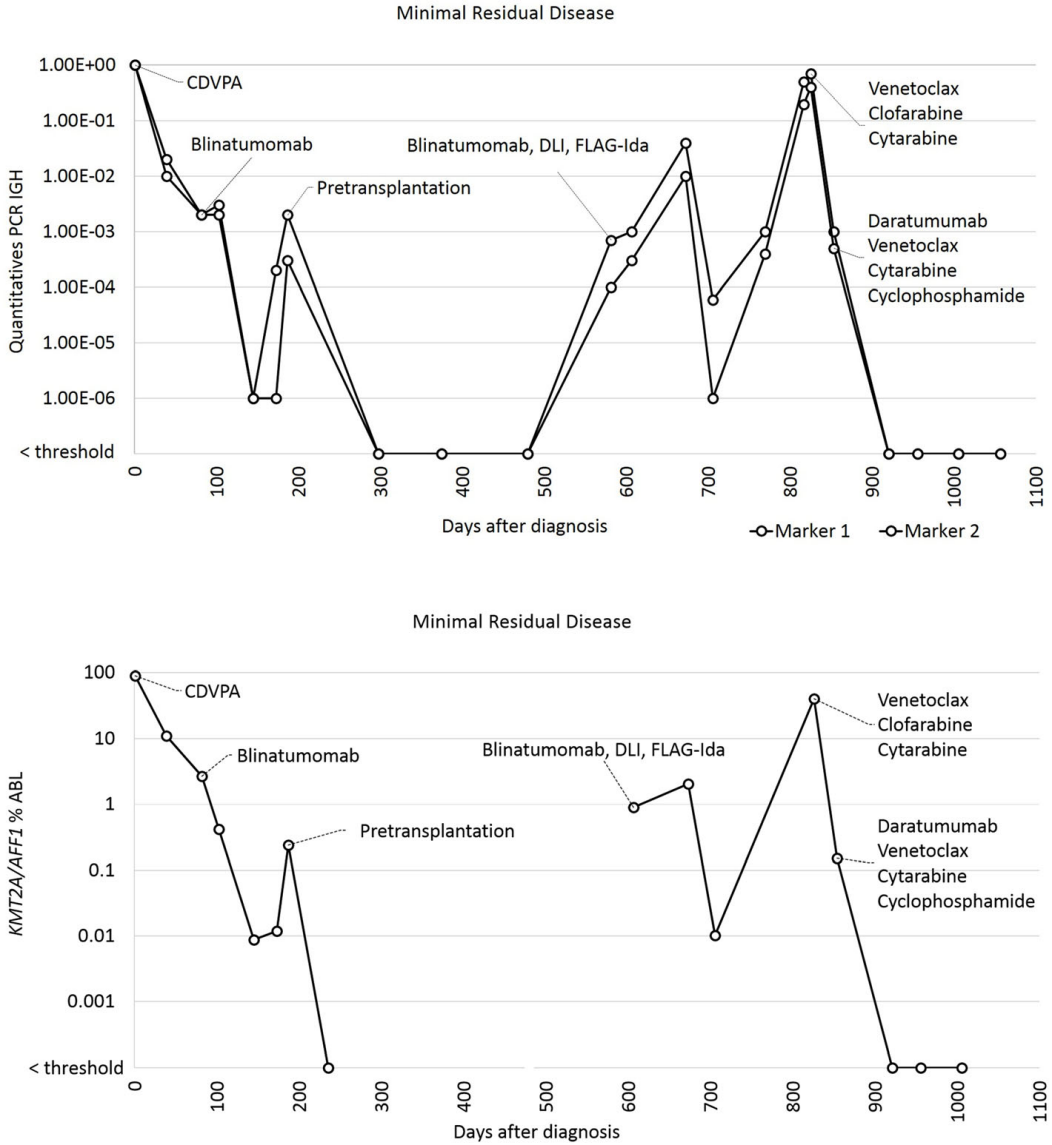
Minimal Residual Disease measured by real-time quantitative PCR: allele specific of immunoglobulin heavy chain (IGH) gene rearrangement (two distinct markers) and (below) : KMT2A/AFF1 fusion transcript (% of ABL reference gene). CDVPA : cyclophosphamide, daunorubicin, vincristine, prednisone, asparaginase.

In the absence of CD19, CD20, and CD22 expression by lymphoblasts, treatment options were scarce in this young adult with ECOG 0 performance status. During stem cell donor search, the patient received clofarabine 20 mg/m^2^/d and cytarabine 1000 mg/m^2^/d for 5 days with venetoclax 400 mg/d (Day1-13), as blasts expressed BCL-2 (Fig. 2.2) and as venetoclax efficacy was reported in pediatric B-ALL models with MLL rearrangements [5, 6]. At Day28, morphologic CR was achieved; however, MRD remained positive (IgH: 10^−3^; *KMT2A-AFF1*: 0.15%).

**Fig. 2:**
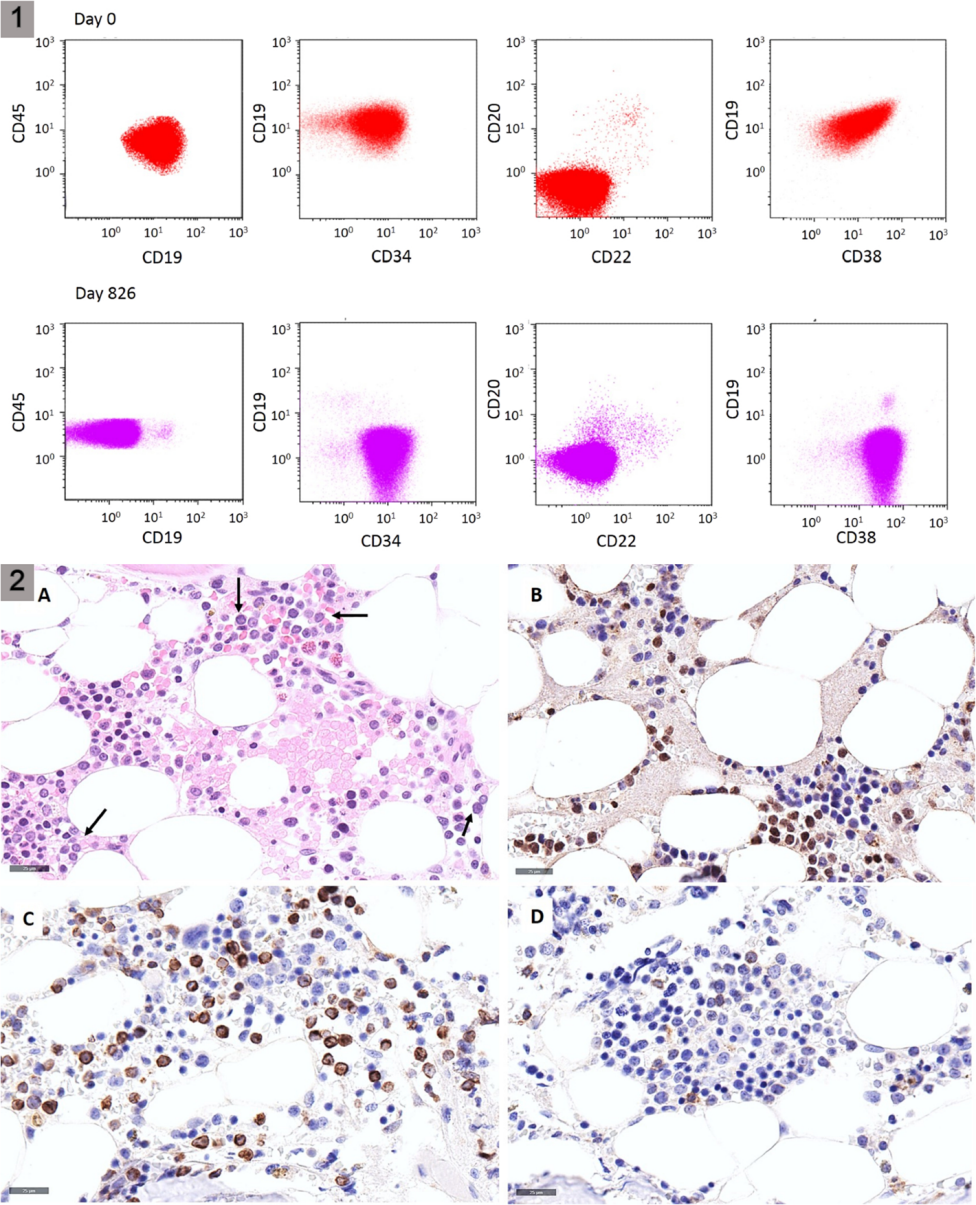
1) Flow cytometry analysis on Day0 showing blasts cells, gated on CD45/side scatter display, are positive for CD19 and CD38 and negative for CD20 and CD22. On Day826, at relapse and after the exposure to blinatumomab, blasts cells have become negative for CD19. 2) Bone marrow biopsy on D826: the bone marrow contained an interstitial infiltrate of blastic cells (arrows) (**A**), which were positive for PAX5 (**B**), BCL2 (**C**) while only a minority were faintly positive for CD19 (**D**).

Considering the potential efficacy of daratumumab in R/R ALL [7–10] and the expression, by lymphoblasts, of high CD38 levels, a therapy targeting both BCL-2 (venetoclax 100 mg, Day3-7) and CD38 (daratumumab, 16 mg/kg, on Day2 and Day10) was administered with cyclophosphamide (150 mg/m^2^ bid Day1-3) and clofarabine (20 mg/m2 Day1-5) to achieve molecular CR. Prolonged agranulocytosis (43 days) was induced with complete hematopoietic reconstitution and molecular CR achieved (sensitivity :< 0.003% *ABL* and <10^−4^ or 10^−5^ for *IGH* rearrangement depending on the marker). MRD remained undetectable during the 5-month delay between the hematopoietic reconstitution and the second ASCT, with a matched unrelated donor. Twelve months post-ASCT, the patient is in molecular CR under venetoclax maintenance with full donor chimerism.

This case illustrates a successful salvage therapy for a very high-risk R/R *KMT2A-AFF1* positive B-ALL, expressing CD38 and BCL-2, but negative for CD19, CD20, and CD22. To overcome chemoresistance, we chose to combine chemotherapy with the BCL-2 inhibitor venetoclax to promote the apoptosis of both lymphoblasts and immature *KMT2A*-mutated leukemia, which may switch to a myeloid phenotype [11]. In order to achieve a deep and sustained molecular remission before ASCT, venetoclax was then combined with the anti-CD38 monoclonal antibody daratumumab, which can induce cell apoptosis via antibody- and complement-dependent cellular cytotoxicity and promote antibody-dependent cellular phagocytosis [12]. Considering the early relapse after FLAG-Ida salvage chemotherapy, the failure to achieve molecular CR with the cyclophosphamide-including induction therapy, and a salvage regimen containing clofarabine and cytarabine; daratumumab and venetoclax most likely played a major role in obtaining the complete molecular response with an undetectable MRD. This hypothesis is further supported by results of ongoing clinical trials and case reports, which show that venetoclax [13] and/or daratumumab [7–10], can eradicate MRD in R/R B-ALL or high-risk T-ALL. This case highlights the importance of the sequential assessment of immunotherapeutic target at the surface of lymphoblasts during the course of treatment, and of the potential role of targeting BCL-2 and CD38 antigen in R/R B-ALL, in particular after CD19 antigen loss following CD19-targeted immunotherapies.

## Supplementary information

Additional file 1: **Supplementary material.** Bone marrow aspiration at diagnosis (A+B), first relapse (Day 673; C+D) and second relapse (Day 826; E+F). MGG staining x 100 (**A, C, E**) and x 400 (**B, D, F**).

## Data Availability

Not applicable.

## Abbreviations

R/R: relapsed/refractory
B-ALL: B-cell acute lymphoblastic leukemia
ASCT: allogeneic hematopoietic stem cell transplantation
PCR: Polymerase chain reaction
CR: complete remission
MRD: minimal residual disease
IGH: immunoglobulin heavy chain
DLI: donor lymphocyte infusion
