# Supplementary figures and images for: Daratumumab and venetoclax in combination with chemotherapy provide sustained molecular remission in relapsed/refractory CD19, CD20, and CD22 negative acute B lymphoblastic leukemia with KMT2A-AFF1 transcript
Source: Biomark Res. 2021 Dec 20;9:92. doi: 10.1186/s40364-021-00343-3 (PMC8686620; doi:10.1186/s40364-021-00343-3)

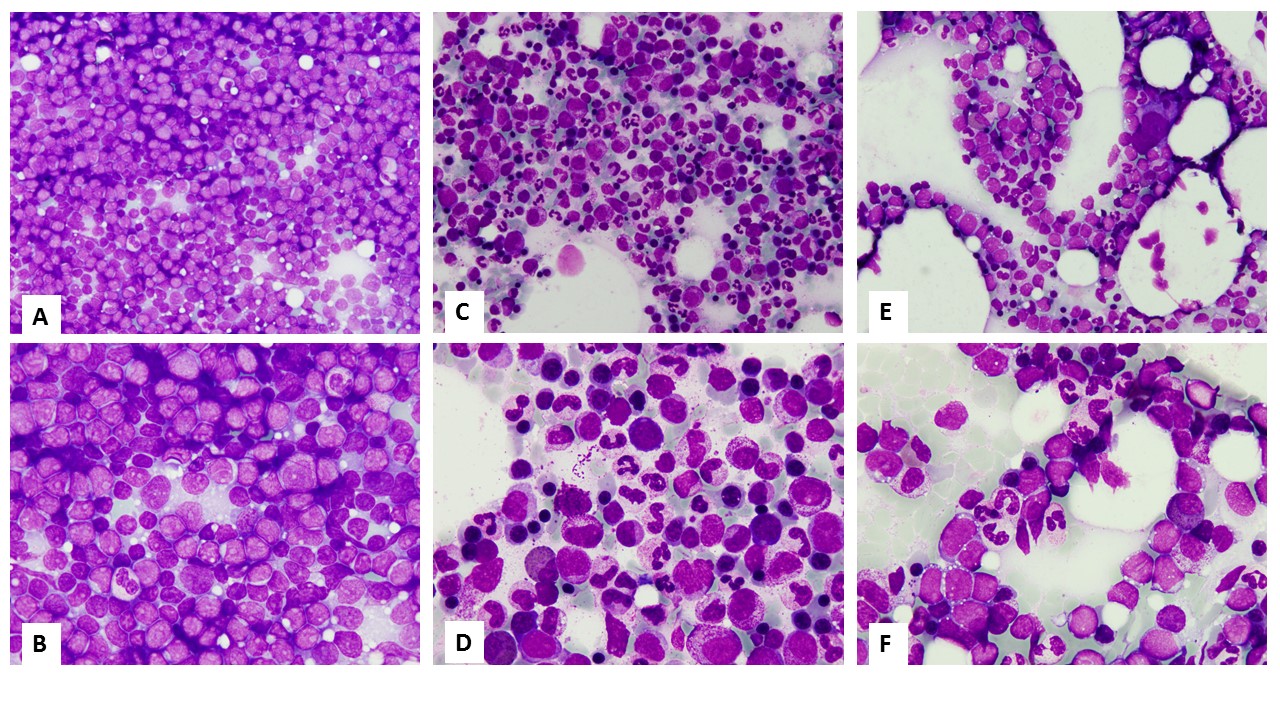

Supplement: Supplementary file 1 — Supplementary material. Bone marrow aspiration at diagnosis (A+B), first relapse (Day 673; C+D) and second relapse (Day 826; E+F). MGG staining x 100 (A, C, E) and x 400 (B, D, F). [file 40364_2021_343_MOESM1_ESM.jpeg]
